# Modal testing for model validation of structures with discrete nonlinearities

**DOI:** 10.1098/rsta.2014.0410

**Published:** 2015-09-28

**Authors:** D. J. Ewins, B. Weekes, A. delli Carri

**Affiliations:** Department of Aerospace Engineering, University of Bristol, Bristol, UK

**Keywords:** nonlinear, modal testing, model upgrading, model updating, model validation

## Abstract

Model validation using data from modal tests is now widely practiced in many industries for advanced structural dynamic design analysis, especially where structural integrity is a primary requirement. These industries tend to demand highly efficient designs for their critical structures which, as a result, are increasingly operating in regimes where traditional linearity assumptions are no longer adequate. In particular, many modern structures are found to contain localized areas, often around joints or boundaries, where the actual mechanical behaviour is far from linear. Such structures need to have appropriate representation of these nonlinear features incorporated into the otherwise largely linear models that are used for design and operation. This paper proposes an approach to this task which is an extension of existing linear techniques, especially in the testing phase, involving only just as much nonlinear analysis as is necessary to construct a model which is good enough, or ‘valid’: i.e. capable of predicting the nonlinear response behaviour of the structure under all in-service operating and test conditions with a prescribed accuracy. A short-list of methods described in the recent literature categorized using our framework is given, which identifies those areas in which further development is most urgently required.

## 1. Introduction

This is an Opinion Piece paper, proposing an approach to a practical problem in an era of growing concern and interest in nonlinear effects from an industrial perspective. The paper begins by defining the class of problem which is being addressed here as this is a particular subset of cases drawn from the whole nonlinear structural dynamics landscape. We are considering the problem faced by industrial developers of critical structures—ones where structural integrity is the overriding structural dynamics concern—in which the current bounds of analysis and testing imposed by assumptions of linearity are challenged and often breached at the edges of the performance envelope. Essentially, if a structure is observed to be operating under conditions where nonlinear effects become a controlling factor in determining dynamic response levels—and thus life, or reliability—how can this best be managed?

### (a) Starting point

There is currently much interest and activity seeking to gain an improved capability to predict the vibration behaviour of systems and structures which contain non-trivial nonlinear features. Much of this work is already quite advanced and addresses many now well-known complex phenomena in nonlinear system dynamics including: bifurcations, jumps, instabilities, chaos, etc. As such, it represents a major change of approach in the modelling and testing of the relevant structures, and this can present a formidable challenge to industrial and other applied users of modern structural dynamics technology. For many of these users, the current state of the art is a highly developed linear modelling, analysis and testing technology which delivers perfectly acceptable results in the majority of applications. ‘Perfectly acceptable’ here means that the dynamic response predictions provided by the available model are within the required tolerance of accuracy that must be prescribed in every application. This is especially important in cases of critical structures where structural integrity is the primary design constraint which must be managed for regulatory as well as commercial requirements.

### (b) The supposed problem

Today, we find that there is an increasing incidence of conventional, essentially linear, models failing to deliver the necessary reliability of response prediction, because of the existence of some nonlinear features in the structure. It is *supposed* here there many of these ‘new’ cases fall into a category where the nonlinearities are localized and are relatively sparse in terms of the number of spatial degrees of freedom (DOFs) in the model that are involved. It is *not supposed* at this stage that these nonlinear effects are weak, or smooth—just that they are localized (discrete) and relatively few in number.

Some of the most common causes of this nonlinear behaviour are associated with the joints used to connect the primary components of the structure under investigation. These joints can be very complex, with features involving friction, stick–slip behaviour, ill-defined static contact stress fields, gaps and so on, and the modelling of their dynamic characteristics is a daunting prospect. The fundamental understanding of the dynamic behaviour of joints is the subject of an ongoing international research effort,^1^ and it is becoming clear that for engineering applications such as are the subject this paper, some form of simplified model is required. Here, it is *supposed* that a low-order approximate model, perhaps a polynomial with just two or three coefficients, might be a necessary compromise to capture the essential nonlinear features—as far as vibration response to external excitation is concerned—at least as a first approximation.

It is *further supposed* that in many of these cases, although not necessarily all, the nonlinearities only become significant in terms of their consequences on the predicted response when the amplitudes of excitation and response increase from the typically low levels that are tested in the laboratory to the generally much higher levels that are experienced under real or simulated operating conditions. The current state of the art in industries that have to address these problems is such that a preliminary model should be expected to have been validated for the lower vibration levels that would be experienced under typical modal testing carried out for model validation.

Lastly, it is *supposed* that an enhanced modal testing and analysis procedure might be a suitable way forward to resolve the limitations in such a design verification procedure that are introduced by the emergence of nonlinear effects in the structure causing an unacceptable reduction in the quality of the model’s prediction. The approach proposed here is to seek evolutionary developments in modal testing and associated analysis techniques that would allow the enhancements of an initial model, validated for low-level response prediction, to provide the same degree of accuracy in predictions of response at higher, operational, levels.

### (c) The proposed approach

The essential requirement is for a procedure which allows the validation of a model that is capable of predicting the structural response for any required operational conditions. It is not concerned with how that response is most efficiently computed (i.e. the algorithm), or what the finer points of the dynamics (bifurcations, chaos, etc.) might be: it is primarily concerned with the construction of a model capable of producing representative response predictions.

In essence, the proposed methodology comprises a set of steps which follow directly from those of the current state of the art for structures, where nonlinearity is not an issue—and so we start with those steps and continue into the new regime, seeking to require as few extra capabilities as possible. The ten steps are grouped into three phases.

#### (i) Phase I preparation

Implementation of a conventional (linear) model validation procedure on the test structure, usually at low levels of vibration, and a preliminary examination for early signs of nonlinear behaviour.

(1) Preliminary modal test for model validation used to detect early signs of nonlinearity.
(2) Low-level modal test to validate an Underlying Linear Model (ULM).^2^
(3) Specification of the representative level test programme.

#### (ii) Phase II test and identification

Conduct further measurements under more closely controlled vibration levels than are normally applied. From these results, ascertain whether the degree of nonlinearity is such that an enhanced model should be developed. If this is positive, conduct an enhanced modal test (Modal Test+) with a view to identifying (i.e. characterizing, locating and quantifying) the main nonlinear elements in the structure.

(4) Modal Test+ Detection (D): to determine strength of nonlinearity effects on response characteristics.
(5) Modal Test+ Characterization (C): to determine character of nonlinearity.
(6) Modal Test+ Location (L): to locate regions containing nonlinear features.
(7) Modal Test+ Quantify (Q): to quantify nonlinear features.

#### (iii) Phase III verification and validation

Seek to upgrade^3^ the Preliminary Model—the ULM—to include sufficient parameters to render model updating a possibility.

(8) Upgrade the Preliminary Model to include nonlinear elements.
(9) Update the Upgraded Nonlinear Model.
(10) Verify the Final Nonlinear Model.

**Figure 1.**
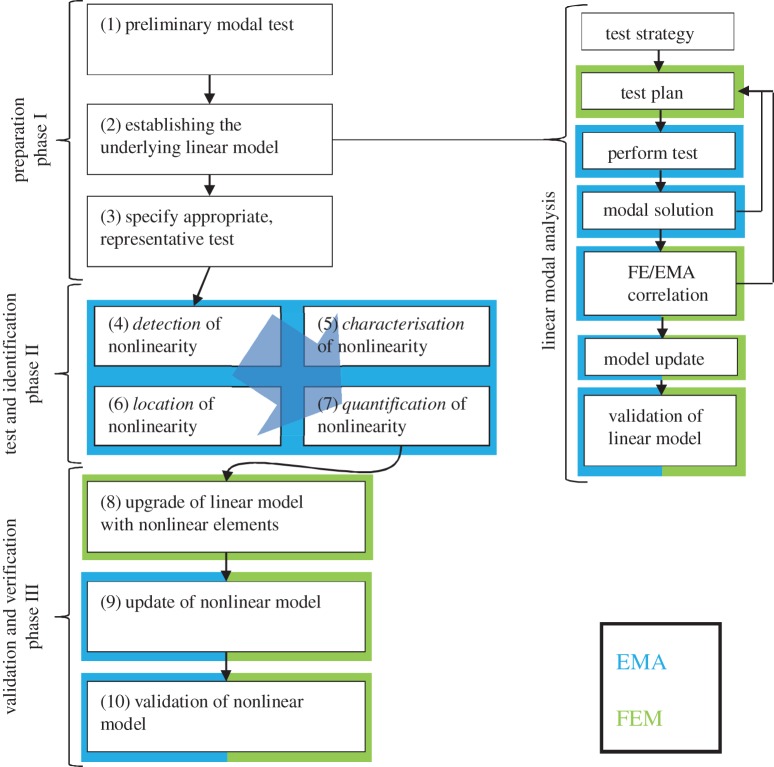
Schematic of the proposed Modal Test+ procedure.

See figure 1 for a schematic of these steps.

As will be seen below, the individual steps require an increasing advancement of data measurement and analysis procedures, over and above those in current modal testing practice, but the approach seeks the minimum advance (and added cost and complication of implementation) which is consistent with achieving the design specification for accuracy of response predictions.

## 2. Essential features of the proposed procedure

In this section, we spell out in more detail some of the issues that will be encountered when carrying out the several steps listed above that make up the overall procedure.

### (a) Phase I preliminary modal test and preparation for nonlinear identification (Steps 1–3)

Some careful preparation is appropriate before embarking on an investigation into the nonlinearity of a test structure. Much of this is conventional modal testing activity but it is worth noting the specific issues to be covered. It is supposed that the first indications that trigger the prospect of undertaking a nonlinearity investigation will come from the data obtained in an otherwise routine model validation procedure. Some inconsistencies in the measured data, or difficulties in deriving unambiguous modal properties from the modal analysis stages, can be early indictors of a degree of nonlinearity that is at the very least interfering with the current validation process, if not contaminating it. Confirmation of such minor inconsistencies is usually the starting point for the procedure outlined here (Step 1).

The next stage (Step 2) is to conduct a controlled model validation exercise aimed at validating the model at a consistently low level or amplitude of vibration. Many practical nonlinearities exhibit a degree of amplitude dependence and most of these become more prominent at higher levels of response, rather than at lower levels. There are some exceptions, as always, and these are often related to loose joints where there are clearances or gaps which are only closed up once the vibration level has reached a certain magnitude. Such gaps are not intended to be active during operation of the structure, and so it may be appropriate to block them with wedges in testing. For the most part, however, the controlled level validation test would be undertaken at the lowest amplitude of vibration that can still provide valid and reliable data—clear of noise contamination.

The idea of this step is to collect data and then use it to validate the model so as to obtain the ULM for the test structure: the best estimate that can be obtained for the near-zero amplitude vibration conditions. This level of response is likely to be one or two orders of magnitude lower than the levels the structure will experience in service.

The last preparatory task (Step 3) is to ascertain realistic estimates of the amplitudes of vibration or excitation forces that the structure is likely to experience in service as these are the conditions for which the ‘new’ representative model is required to be applicable, or ‘validated’. It is necessary to determine these conditions with some accuracy in both level and spatial distribution of applied excitation forces, as these conditions need to be reproduced in the advanced modal test (Modal Test+) that will be conducted to explore the nonlinear features. These details are then used as the basis of the test planning for the Modal Test+.

### (b) Phase II identification of the nonlinearity (Steps 4–7)

This section contains the majority of the proposed new procedure and comprises four stages to complete the identification adequately for the purpose of validating a model to describe the nonlinear effects. The four stages are: (i) Detection, (ii) Characterization, (iii) Location, and (iv) Quantification (DCLQ). These stages have been recommended and quoted before in [1], but here we seek to establish how to carry them out and how to use the results for the strategic objective of achieving a validated model.

**Figure 2.**
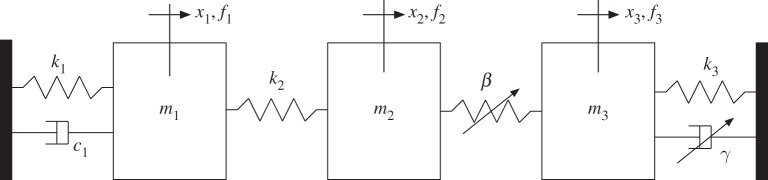
Representative 3DOF system with discrete nonlinearities.

At this stage, it is perhaps helpful to introduce a conceptual description of the kind of characteristics that we are aiming to identify. We can illustrate the basic concepts using a very simple 3DOF system (figure 2), whose equations of motion might have a form similar to the following:
2.1
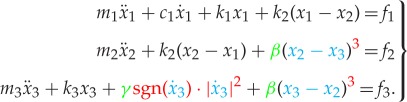
It is clear from the above equation that the system is nonlinear due to the presence of displacement and velocity terms that behave in a nonlinear fashion (non-unit exponents, i.e. higher order terms). These terms (coloured in equation (2.1)) are also known in the literature as ‘nonlinear restoring forces’, because they are often moved to the r.h.s. of the equations and treated as forces that keep the system balanced.

It should be noted that even if the first equation in (2.1) appears to be linear, mass *m*_1_ experiences some effect of the nonlinearities in the system through its connection to mass *m*_2_, so there is no distinction between ‘linear’ DOFs and ‘nonlinear’ DOFs. It is only possible to recognize ‘actively nonlinear’ DOFs from ‘passively nonlinear’ DOFs.

By analysing each of these nonlinear terms in detail, we recognize three main features:

(a) the presence of a nonlinear functional *g*(⋅)—in red in equation (2.1),
(b) the variables on which this functional acts—in blue in equation (2.1), and
(c) scale factors—in green in equation (2.1).

The nonlinear functional and the *types* of variables on which it acts (displacements, or velocities) are properties that give ‘character’ to the nonlinear term, and so we refer to them as ‘characteristics’. In the case of equation (2.1), we can say that the nonlinear spring has a ‘cubic stiffness’ (i.e. a monomial^4^ of order 3 that acts on displacements) while the nonlinear dashpot is a ‘quadratic damper’ (i.e. a monomial of order 2 that acts on velocities). The identification of these properties is here addressed as *Characterization* of nonlinearity.

The DOFs related to the quantities on which the nonlinear functional acts define the ‘location’ of the nonlinear elements. We can distinguish ‘grounded’ nonlinearities from ‘non-grounded’ nonlinearities by looking at how many DOFs are involved in defining the nonlinear functional variable, so in equation (2.1) the spring is a non-grounded nonlinear element (acting between DOFs 2 and 3) while the dashpot is a grounded nonlinear element (acting between DOF 3 and the ground). The identification of this property is hereby addressed as *Location* of nonlinearity.

The scaling factor of each nonlinear term is there to define its strength in the overall equation. If the strength of the nonlinear terms is much lower than of their linear counterparts, it is safe to assume that the nonlinearity can be neglected or absorbed in the linear terms (*linearization*), while if the strength of the nonlinear terms is comparable to, or even greater than, those of the linear ones, then non-trivial nonlinear effects are expected. The identification of the scaling factors (also referred to as ‘coefficients’) is hereby addressed as *Quantification* of nonlinearity.

At this stage, it is appropriate to draw attention to the important distinction between what we refer to as *parameters* and *coefficients*. *Parameters* are part of the Characterization process (e.g. the polynomial exponents such as cubic or quadratic terms are parameters) while *coefficients* are simply scaling factors of the nonlinear terms, describing their strength in the equations.

#### (i) Detection of the nonlinearity (Step 4)

In this and the following three subsections, we provide a brief definition of the relevant step and its specific objectives, followed by a discussion of the main issues that will be faced in carrying it out.

Here, *Detection* of nonlinearity is used to indicate that some effect attributed to nonlinearity is observed, and it is deemed that the system response cannot be adequately represented by a linear model. This triggers further investigation to obtain more detailed information from the tests in order to be able to upgrade the model with nonlinear terms. This upgraded model can then be updated with the full set of identification data. The Detection step should yield the following insights:

(i) the presence of any nonlinear effect(s) and
(ii) optionally, the relative strength of the nonlinear term(s) with respect to the linear terms.

The detection of nonlinearity is the first step in determining whether and—if yes—to what extent a linear finite-element (FE) model must be upgraded in order to capture the relevant dynamics of the associated real structure. Tests to detect nonlinearity should be performed at relevant input forcing levels (i.e. representative of operating service conditions). The detection of nonlinearity would ideally be performed from response data, since it is response characteristics that ultimately are the targets which the validated model must match, i.e. if the response data can be closely approximated by a parsimonious linear model then this would be acceptable for the scope of systems considered here, and nonlinear identification need not be progressed further. The non-detection of nonlinearity can be seen as a form of validation of the use of linear theory and models, although caution is required because the non-detection result could also be a false-negative from using deficient methods for detection of nonlinearity. A positive detection of nonlinearity indicates the need for nonlinear terms to be included in the equations of motion, such as indicated by the coloured coefficients in equation (2.1). A false-positive detection (e.g. due to noise) is undesirable because the additional test and model complexity is a significant cost and undertaking.

The primary objective of the Detection phase is to ascertain the presence of a nonlinear feature in the structure which requires enhanced representation in the equations of motion of the model (equation (2.1)). A secondary, optional, objective of the detection stage is to determine the ‘strength’ or severity of the nonlinear effect, based on the importance of the nonlinear terms in the model with respect to the linear terms of the original model. It might be convenient to envisage four levels of nonlinearity severity, *trivial*, *weak*, *mild* and *strong*, corresponding to different levels of subsequent action in terms of model upgrading, such as:

— trivial=can be neglected;
— weak=can be linearized: a linear model can be developed to generate acceptable response data for a specific operating envelope;
— mild=must be accounted for; and
— strong=must be accounted for, and may require a major enhancement of the original model and its subsequent response analysis.

There might also be a good case for seeking to attribute some quantitative indicator to each of the nonlinear Detection methods, to help indicate the appropriate category for each new structure submitted to this proposed procedure. There are several such indicators that are used to ‘measure’ the deviation of a prescribed set of points from an anticipated baseline, and these could also provide some means of comparative measure of the severity of each nonlinearity. It is considered to be beyond the scope of this paper to postulate such an indicator, but it is appropriate to note that one may well be necessary when these procedures become more widely used.

#### (ii) Characterization of the nonlinearity (Step 5)

*Characterization* refers to the physical origins of the individual nonlinear elements and is primarily concerned with the identification of whether it is the stiffness characteristics that are the main source of nonlinearity, or the damping terms, or both. A secondary objective of this step is to establish the approximate order of the nonlinear feature, primarily to assist in deciding how many additional coefficients will be required in the model upgrading process, but also to aid understanding of the basic physical origins of the nonlinear behaviour of the properties that give ‘character’ to a nonlinearity.

A Characterization step should yield the following insights:

(i) which aspect(s) of motion drive the nonlinear behaviour (e.g. displacement, velocity);
(ii) how the nonlinear effect might need to be described functionally (e.g. polynomial, multi-linear); and
(iii) what are the parameters of the functional (e.g. exponents of polynomial).

This Characterization step seeks to establish the underlying nature of the nonlinearity, and it is important as much for the physical understanding of the behaviour as for the quantitative data. In effect, this means establishing whether it is the stiffness elements which are nonlinear, or the damping or even the inertia features—or any combination of all three. Further, the aim is to establish the form of the deviation from linearity, e.g. typically, hardening or softening for stiffness elements, and increased or decreased damping for damping elements. A convenient mathematical function to describe the nonlinearity is sought, and will often comprise polynomial terms for mathematical convenience. These forms will act upon the absolute displacement, velocity or acceleration of a single DOF, or between the relative displacements, velocities or accelerations of a pair of DOFs, which for a polynomial-type nonlinearity sees these quantities raised to a non-unity index. For the purposes of Characterization, the index is suspected without necessarily knowing to which DOFs it applies, i.e. the red term in equation (2.1) is to be found, while the blue terms are not yet known.

Note that for the purposes of FE modelling, it is likely that the nonlinearity will be characterized using convenient mathematical functions, but some methods of characterizing nonlinearities yield non-parametric forms, which are superior since no *a priori* model is implied by non-parametric characterization methods. A parametric form can be fitted to the non-parametric characterization, and the specific parametric form evaluated for quality of fit. This step, and the previous one, can be undertaken with relatively few measurements, because at this stage the identification is not spatial. This is believed to be one of the most important parts of the whole identification process (considered as DCLQ altogether) because it directly informs how to *upgrade* the FE model.

#### (iii) Location of the nonlinearity (Step 6)

*Location* refers to determining exactly where the nonlinear features are located in the spatial description of the structure. In mathematical terms, this means identifying which of the model’s DOFs are closely located to the source of the nonlinear behaviour (e.g. the ones across a joint).

A Location step should yield the following insights:

(i) how many nonlinearities are present;
(ii) which DOFs are involved in the description of each nonlinear effect; and
(iii) the precise configuration, or connectivity, of the nonlinear features (i.e. grounded/non-grounded).

This step builds on the previous two in seeking to locate where in the structure the nonlinear elements are to be found, while the primary objective is to identify which DOFs in the model must be included in the model upgrading process. It can also serve the useful purpose of guiding the selection of measurement sites for use in the upgrading and updating processes in order to gain maximum information regarding the specifics of the elements of interest.

Location of the nonlinearities is an essential prerequisite for introducing additional parameters in the multi-DOF models and, within the scope of this document, this is limited to discrete nonlinearities (i.e. each nonlinearity can be isolated between elements, or between an element and ground). The Location step of nonlinearity identification gives us the blue terms in equation (2.1).

Location might be considered as part of the Characterization step, although methods for locating nonlinearities are actually a limited subset of the methods capable of characterization (methods which offer location tend to also offer characterization). We observe that while there are some methods for characterizing nonlinearities using standard commercially available equipment and software, none of these systems are capable of locating the nonlinearities.

#### (iv) Quantification of the nonlinearity (Step 7)

In this step, *Quantification* refers to the search for the correct numerical values of each coefficient associated with a previously characterized nonlinear term.

The Quantification step should yield the following insight:

(i) coefficients (scaling factors) for the functional forms that characterize the nonlinear effects.

Methods for quantification of nonlinearity give the effective strength to the characteristic nonlinear functional form, i.e. a coefficient value such as the green term in equation (2.1). Methods which quantify do not necessarily detect nonlinearity, although detection may be inferred through the coefficient value itself (depending on whether it makes the associated nonlinear term significant compared with the linear terms in the equations of motion within the relevant operating range, or indeed variance in predicted coefficients for the nonlinear terms). The same argument for detection of nonlinearity by quantification can also be made for characterization and location, but will typically require extensive computation and may not result in unambiguous identification.

In some situations, the Characterization step can deliver sufficiently ‘good’ results that Quantification can be performed within Model Updating, i.e. the initial (pre-update) coefficient need only be nominally established.

### (c) Phase III upgrading, updating, verification and validation of nonlinear model (Steps 8–10)

The final phase in this process involves one new task and two traditional ones—8, 9 and 10, respectively. Model Updating (Step 9) is quite a well-developed procedure now for linear systems, and there are a number of algorithms and software packages that can be used to support this task. However, there is an important qualification that applies to all updating exercises, including for linear structures, and that is the need to verify that the model is capable of being updated before embarking on that process. Simply, this means ensuring that the model does in fact include sufficient parameters or elements to be capable of describing the structural behaviour adequately once the correct values for these parameters have been determined. The problem that can arise is that a model updating procedure can be applied to a model which is not verified, and while a result is obtained, this result is unreliable, and often involves the adoption of unrealistic values for the parameters that have been updated.

In the case of a nonlinear structure, a new issue arises in the sense that each model element is likely to have a higher order characteristic than would be the case for a linear structure. In other words, an individual stiffness element might require twice as many (or more) parameters to be included to describe the nonlinear physical behaviour accurately enough. To accommodate this possibility, and to ensure a verified model being available for the final stage of updating to determine the correct numerical values of all the crucial elements, the process of establishing how many, and which, parameters are necessary for the model to be capable of describing the relevant physical behaviour with the desired accuracy is defined here as *Model Upgrading* (Step 8). In effect, this same process can be used in both linear and nonlinear systems: ensuring that the model being validated has sufficient tuneable parameters to allow an effective updating to be performed applies in all cases. In recent applications for linear model validation, this phase has sometimes been referred to as ‘verification’ of the preliminary model but is perhaps better described by the new definition of ‘upgrading’. Once this condition is satisfied, it is then possible to proceed with the classical model updating process, which is concerned with determining the correct values to assign to each of the coefficients in the model (Step 9).

One final check that can be done to complete the whole procedure is to verify the final model by using it to predict a new response characteristic and then to perform a measurement to demonstrate that the prediction is sufficiently accurate (Step 10).

## 3. A brief overview of current identification techniques

### (a) Introduction

In the previous section, we described our proposed procedure for managing situations where a tested structure has nonlinear features which have non-negligible influence over its dynamic response levels, and thus its life or reliability. We now turn attention to the testing and analysis tools that are required to carry out these procedures. We have included a number of references (table 2) which relate to some methods currently under development that have the potential to provide the means of carrying out the steps that we have listed above. We are conscious that we have included a relatively small number of references, but the methods listed comprise those commonly discussed in the literature, and some lesser discussed methods which yield direct physical insights. A more comprehensive list of the earlier references is included in [1]. It is not our intention to provide a critical review of these methods, but rather to illustrate the general level of current capability in this area, and to identify the further advances that are necessary for the procedures proposed in this paper to be carried out in practice. In the following paragraphs, some comments are made on the current state of the art in all steps, but particularly in Steps 4–7, which is the core topic of interest here. The references are not identified individually in the text, but a summary of their applicability to the core identification tasks is provided in tabular form in appendix A.

### (b) Comments regarding measured data in modal tests

As mentioned in the previous sections, the assumed starting level is the availability of state-of-the-art modal testing and analysis capabilities, together with suitable means of carrying out model updating—all within the confines of linear structural dynamics behaviour. These capabilities are necessary and sufficient for carrying out Steps 1–3 of the 10-step procedure listed in §2. No references are given for this phase, but the following comment is made in respect of the transition from linear to nonlinear domains and, in particular, to the widespread use of frequency response function (FRF) properties as the primary visual representation of the dynamic response behaviour of structures of all types. While the actual data measured in a modal test are invariably time histories of responses and input excitations, these need to be presented in some form of response function (response per unit input) for the purposes of interpretation or modal analysis, and the FRF or its time domain equivalent—the impulse response function—are the two prominent options. In practice, it is normally a set of FRFs that are the outcome of the measurement phase. Seeking to extend our modal testing techniques into the nonlinear domain presents an immediate difficulty because, strictly speaking, the classical FRF does not ‘exist’ in these circumstances. Rather, it is strictly necessary to define a more rigorous and complex function to capture the ‘response function’ of a nonlinear system, but this will involve an immediate expansion of the measurement process which we are seeking to delay as long as possible in our pragmatic approach to extend conventional modal testing techniques into the nonlinear domain.

It is well known that if one carries out a series of alternative excitations (periodic, random, transient) to measure the response characteristics of a nonlinear structure, then—unlike the expectation for a linear structure—we do *not* obtain the same result in each case. Nevertheless, it *is* possible to carry out an FRF measurement using any of the standard excitation signals, and to process the measured data in exactly the same way as is normal for linear structures, and then to obtain a set of ‘FRF’ measurements for the nonlinear case. It is known, of course, that these FRF data are deficient; essentially, because we have not included all the information which has been generated in the measurement process by selectively ignoring or averaging components in the response data which are generated by the nonlinearity of the test structure. Simply, if we excite a nonlinear structure with a pure sine tone excitation, then the response will generally contain a component at the excitation frequency (as normal) plus additional components at other (typically higher) frequencies. The extra information contained in those higher components comes from the fact that the nonlinear structure has a higher order behaviour than the linear one (for example, a cubic component in the stiffness) and so cannot be completely described by the conventional single-term FRF format. Depending on exactly which signal is used for the excitation, this extra information can be eliminated or subsumed into the overall FRF according to the algorithm used to compute the response function. Further discussion on this issue can be found in [2–4].

It is strongly recommended that whenever there is reference to an FRF measurement on a nonlinear structure, it should be accompanied by a clear qualification of the signal type used for the excitation, in addition to the strength of the input forcing.

### (c) Methods for nonlinear identification (Phase II: Steps 4–7)

A large number of methods for nonlinear identification can be found in the literature, and many of these continue to be developed. However, it is not our intention here to provide a critical review of these methods: for this we refer the reader to [1]. In this section, we classify the families of nonlinear identification methods in a way that guides their use within the identification scheme detailed in §2b, but without the requirement for comprehensive knowledge of all methods (or at least permitting short-listing candidate methods for further study).

**Figure 3.**
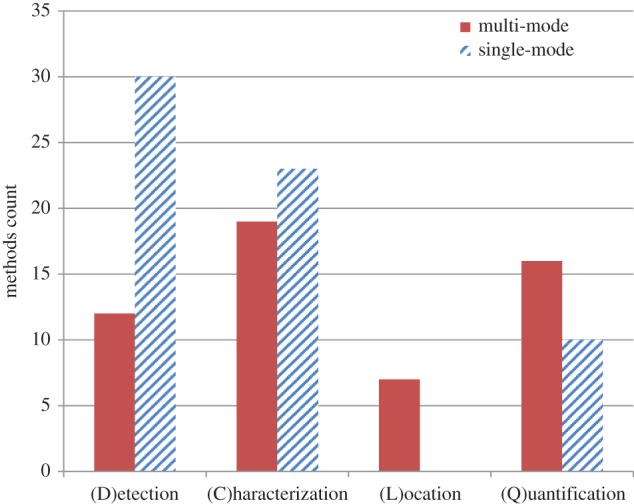
Methods for nonlinear identification available (including those requiring bespoke development), subcategorized by Detection, Characterization, Location and Quantification, and by single or multiple DOF ability.

We have undertaken a survey of the nonlinear identification methods discussed in the current literature—both methods for which development appears to have slowed, and methods still under active development—that may usefully extend the state-of-the-art modal testing capability in the direction we are proposing here. These methods have been categorized into the four core identification stages of Detection, Characterization, Location and Quantification of discrete nonlinear features in the structural model (Steps 4–7), and the most common methods suited to the scope of nonlinear systems considered here are listed in appendix A. In addition to our core definitions for the identification abilities given in §2, we added further ‘indirect’ identification methods for Characterization and Quantification: see table 1 in appendix A for these definitions. Note that the definitions of ‘direct’ and ‘indirect’ methods for classification of nonlinearity are biased towards upgrading and updating an FE model, which is intrinsically a meaningful model comprising physical parameters. As such, nonlinear identification methods which directly consider or yield physical parameters are considered preferentially, although less direct strategies may nonetheless deliver a sufficiently ‘good’ nonlinear FE model in some cases (albeit, perhaps, at greater computational cost or reduced confidence). The count of methods in the review categorized by these identification abilities is given in figure 3, subdivided into methods which are (i) primarily single-mode based and (ii) those which consider multiple modes. These review data given here are a snapshot of what is hoped to become an ongoing process, undertaken as part of the EPSRC ENL Programme Grant.^5^

#### (i) Detection (Step 4)

The Detection step establishes whether a nonlinear model is necessary, and this decision is ideally made quickly and unambiguously using the equipment available in a standard linear modal test. Somewhat unsurprisingly, given the ubiquity of the FRF, methods for detection of nonlinearity based on characteristic distortions of FRF plots make up the majority of the Detection (and indeed Characterization) methods counted in figure 3. There remains work to be done on confidence in detection/non-detection of nonlinearity based on distortion of FRFs, but these methods are appealing because they can largely be performed using conventional equipment. The distortions of the FRFs are also somewhat self-evident without prior knowledge of the specific system, in the sense that deviation from linearity is directly shown by the shape of the resonant peaks (i.e. their deviation from the classical form). Note that while commonly employed for this role, coherence functions are only indirect indicators of nonlinearity.

Direct signal inspection is another possibility using conventional equipment, and is very direct and can be insightful. Input waveforms need to be simple if the output (or indeed reference input) data are to be directly understood, and some enhanced analysis tools (data cursor, reference functions) aid this data inspection. Time–frequency analysis in the form of spectrograms is another relatively simple and useful method, but is not a common facility in standard commercial packages.

In addition, there are a number of other methods such as those in the modal domain, which are arguably unnecessary for Detection, and are ‘indirect’ compared with the above methods—i.e. methods which give a relatively direct representation of the data such that deviation from linearity can be directly observed are preferred, especially the FRF-based methods. A special mention is given here to force appropriation methods such as nonlinear normal modes, because the force appropriation procedure directly detects the presence of nonlinearity at the test stage, as validated by a non-trivial correction applied to the input forcing to achieve excitation of individual mode shapes. This is also apparent in the analysis, where separation into independent modal components is observed to fail (i.e. ‘modal interaction’). However, the specific test requirements for force appropriation are unlikely to be used for the Detection step unless non-trivial nonlinearity is strongly suspected *a priori*.

In summary, Detection of nonlinearity is a well-established practice through many FRF-based methods using standard equipment. Direct signal time history analysis is also possible and, in limited cases, time–frequency analysis. Detection of nonlinearity is also possible using some more advanced analysis methods, but is only likely to be available in cases where detection has already been achieved or pre-assumed, and therefore further nonlinear analysis has already been warranted.

#### (ii) Characterization (Step 5)

The FRF, time domain and time–frequency domain methods discussed for the Detection phase can also give some Characterization information, since the preferred detection methods require minimal manipulation of the data, and the prior Detection of the nonlinearity was therefore dependent on fundamental observations of nonlinearity. These observations can give the characteristic of the nonlinearity without necessarily giving the associated location or strength. However, while many of these methods require minimal extension of basic data plotting, they are often not natively possible within the commercial packages and require exporting the data (such is the simplicity of many of these Characterization methods that they can be quickly put together within even a simple spreadsheet package). This is perhaps unsurprising since the commercial packages tend to make no attempt to address nonlinearity, reflecting the difficulties involved in nonlinear analysis, particularly in devolving the analysis choices to an end user who may not understand the inner workings of the analysis.

Other methods for Characterization of nonlinearity tend to describe behaviour using complicated algebra, or brute-force trial-and-error computation based in the spatial domain (i.e. considering the equations of motion), thereby avoiding some of the complications arising from the loss of linearity in the FRFs (§3b) and modal space. As such, the characterization yielded by these methods tends to be the best-fit of an *a priori* analytic model (or one of a family of trialled models) and is obtained with some difficulty and expense. These methods are included here primarily as additional means to confirm the character of the nonlinearity (typically, by looking back at the Characterization step from the later Location and Quantification steps which demand such methods). The exception to this category is restoring force surfaces, which can give significant (and, notably, non-parametric) insight into the character of the nonlinearity, but is a typically single DOF analysis.

#### (iii) Location and quantification (Steps 6 and 7)

The existing methods for Location and Quantification of nonlinear features are essentially those first mentioned as ‘other methods’ for Detection and Characterization of nonlinearities in the previous sections. This follows from the fact that these methods are typically based on the trialled parameters or the closest fit to an *a priori* analytic form. However, methods based on FRFs, time-domain or time–frequency representations are of limited use for Location and Quantification given that all DOFs will tend to exhibit some effects from the nonlinearity, and not necessarily in an obvious or intuitive way.

We perceive location of hidden/previously unknown nonlinear features as a greater challenge than the previous steps of Detection and Characterization, largely because the act of trying to locate infers analysis of a multi-DOF system, and any nonlinear system which passed the Detection stage will typically exhibit an influence of the nonlinearity at all DOFs—making standard FRF, time-domain and time–frequency plots of limited use. Indeed, the measured DOFs may be inadequate to be connected meaningfully by a representative nonlinear element.

All known current and commercially available modal testing systems lack any facility for locating or quantifying nonlinearity, with all existing methods requiring some bespoke development. The Location step is, however, often facilitated by engineering knowledge/intuition, since the cause (and therefore location) of the nonlinearity can often be narrowed down to a small number of possibilities—typically, joints—especially for the excitation levels experienced by common engineering structures. Quantification is the least difficult part of the identification process, provided that the Characterization and Location steps have successfully completed. Since the coefficients are only scale factors, one can easily attempt ‘Quantification via FE updating’.

### (d) Upgrading and updating models to accommodate nonlinearities (Steps 8–10)

While this paper is primarily concerned with the specific task of identifying nonlinear features in a particular structure through specific measurement and analysis steps, the successive steps of using this information to upgrade and update a model are of relevance, because there needs to be a validated procedure for introducing a known nonlinear feature into an FE model. The inclusion of nonlinear elements in FE models will need to be the subject of a future paper, but some relevant comments are included here.

*Upgrading* a model requires changes to be made to the equations of motion of a discretized (FE) system in order to accommodate previously identified nonlinear terms, once the relevant terms have been located and characterized. This procedure is straightforward in most commercially available FE packages that allow the user to introduce custom elements (springs, dampers) between two DOFs and to define custom force–displacement/velocity relationships for each of them.

*Updating* involves adjusting the values of the coefficients which have been identified by Quantification in order to model the response characteristics. Updating is an iterative procedure: each updating loop requires the evaluation (simulation) of the modelled system and subsequent correlation with experimental data. If the residual errors are too high, another updating loop is performed, using a different set of initial conditions.

The measured data are usually displayed in the form of FRFs, while—at the present time—the evaluation of the system is generally performed in a time-domain framework. Time-domain simulations are generally very slow due to the small time steps involved and the fact that they must evaluate through all the transient information to get to a steady state. This scales poorly with the dimension of the system and results in a difficult correlation process, requiring extensive resources and post-processing effort in order to trim the transients and cast numerical data in the same domain as experimental data. This heavy post-processing adds to the evaluation time, rendering the updating process unacceptably slow.

Given this issue, there arises the need for a simulation tool that lets the user evaluate the nonlinear equations directly in the steady-state frequency domain. There are already some methods under development, mainly using harmonic balance and numerical continuation techniques [5]. The updating of nonlinear FE models will definitely benefit from these methods, once perfected.

## 4. Prospects

In this paper, we have proposed a methodology for extending conventional modal testing and model validation for linear structures to applications where the test structure demonstrates clear indications of nonlinear features, typical of many practical structural assemblies in industrial practice. The primary objective of the procedure is to construct an enhanced model of the structure which is capable of predicting the structure’s response to a range of dynamic loading and excitation conditions typical of those encountered in service.

The proposed procedure subdivides the primary task of identification of a nonlinear feature into its constituent elements of Detection of nonlinearity; Characterization of the nonlinear elements; Location of these elements within the structure; and Quantification of the individual parameters which define the nonlinear behaviour.

In order to address the extra complications that the higher order models associated with nonlinear behaviour introduce, a new procedure of model *upgrading* has been proposed as a necessary precursor to the model *updating* phase where the most effective numerical values are assigned to each of the model parameters. These two steps are, in effect, verification and validation of the model, respectively.

We have included references describing what we believe to be the most common methods available and under development for nonlinear model identification, and categorized these methods within our identification framework. We have not selected or indicated preferences among the lists of new methods as that would be a step beyond this paper’s purpose. However, we have sought to indicate the requirements of the various identification stages and hope that this will serve to guide and expedite development of the more useful procedures, especially with respect to the industrial needs of pragmatism and realism when dealing with nonlinear structures.

## Footnotes

1 ASME Research Committee on Mechanics of Jointed Structures. See https://community.asme.org/research_committee_mechanics_jointed_structures/w/wiki/3795.activities.aspx.

2 The ULM assumes that the nonlinear effects increase with excitation level, and so at a near-zero excitation the nonlinearities are negligible. Strictly speaking, there are nonlinearities which are only ‘significant’ (in the sense of deviation from linearity) at near-zero excitation levels (e.g. a small clearance), but this sort of nonlinear effect will typically be insignificant at the high excitation levels which are of interest.

3 ‘Upgrading’ a model implies adding parameters to it to make it complete, as opposed to ‘updating’ which involves adjusting the values of parameters that are already included, but may be inaccurate. It is believed that this is the first time this distinction has been formalized, and it takes the place of the references in the literature of it being necessary to *verify* a model before it can be subjected to a model updating procedure. It is critical that all the parameters that need to be updated from a physical reality perspective are actually in the model, and for many years this has not been mandated. Now, because of the added complexity of nonlinear elements requiring higher order terms for their proper description, it must be.

4 N.B. A polynomial nonlinearity can be regarded as a set of monomial nonlinearities that happen to act on the same location.

5 EPSRC Programme Grant, ‘Engineering Nonlinearity’.

## Appendix A

See tables 1 and 2.

**Table 1.** Table of ‘short’ definitions, used in the methods list to characterize the nonlinear identification methods (table 2).

	direct	indirect
detection	the method detects nonlinearity by some self-referencing or consistent means	n.a.
characterization	the method characterizes nonlinearity by giving the stiffness or damping terms in the equations of motion as a closest analytic form, or a (possibly unscaled) non-parametric function	the method can distinguish between stiffness and/or damping nonlinearity
location	the method locates nonlinearity—this is typically between two DOFs, or one DOF and ground	n.a.
quantification	the method quantifies the stiffness or damping nonlinearity by providing scaling for the closest analytic form or a scaled non-parametric function	the method gives a non-physical quantification, which may be used indirectly to update a nonlinear model, e.g. NL ‘mode shapes’

**Table 2.** Abridged table of reviewed major nonlinear identification methods for quick reference. These references are intended to be useful to the reader, rather than necessarily the originator of the method. Note that the DTA Handbook [2], Worden & Tomlinson [3] and the review by Kerschen *et al.* [1] feature prominently as de facto references due to their usefulness and accessibility.

	direct	indirect
detection	FRF distortion methods (various) [2,3]	n.a.
	time-signal inspection (various) [2]	
	CONCERTO [6]	
	higher order statistics/FRFs [2]	
	associated linear equations: SDOF [7], MDOF [8]	
	nonlinear normal modes: FANS [9], NLRD [10], experimental (Shaw–Pierre) [11]	
	restoring force surfaces: SDOF [2,12,13]	
	non-stationary analysis: [14–22]	
characterization	CONCERTO [6]	FRF distortion methods (various) [2,3]
	associated linear equations: SDOF [7], MDOF [8]	time-signal inspection (various) [2]
	inverse harmonic balance [23]	higher order statistics/FRFs [2]
	restoring force surfaces: SDOF [2,12,13], MDOF (direct parameter estimation) [24]	proper orthogonal modes [1]
	reverse path method: standard [25] (and NIFO [26]), conditioned [27], orthogonal [28]	
	nonlinear structural model updating [1]	
		restoring force surfaces: modal space [3]
		nonlinear PCA [29]
		non-stationary analysis [14–22]
		nonlinear normal modes: experimental (Shaw–Pierre) [11]
location	nonlinear output FRFs [30]	n.a.
	inverse harmonic balance [23]	
	restoring force surfaces: MDOF (direct parameter estimation) [24]	
	associated linear equations: MDOF [8]	
	reverse path method: standard [25] (and NIFO [26]), conditioned [27], orthogonal [28]	
	nonlinear structural model updating [1]	
quantification	CONCERTO [6]	restoring force surfaces: modal space [3]
	associated linear equations: SDOF [7], MDOF [8]	nonlinear normal modes: NLRD [10], experimental (Shaw–Pierre) [11]
	inverse harmonic balance [23]	
	restoring force surfaces: SDOF [2,12,13], MDOF (direct parameter estimation) [24]	
	reverse path method: standard [25] (and NIFO [26]), conditioned [27], orthogonal [28]	
	nonlinear structural model updating [1]	
	nonlinear subspace identification [31]	
	non-stationary analysis [14–22]	

## References

[1] Kerschen G, Worden K, Vakakis A, Golinval J 2006 Past, present and future of nonlinear system identification in structural dynamics. Mech. Syst. Signal Process. 20, 505–592. (10.1016/j.ymssp.2005.04.008)

[2] Dynamic Testing Agency (DTA). 1996 Handbook on guidelines to best practice in dynamic testing. Cranfield, UK: DTA.

[3] Worden K, Tomlinson G 2001 Nonlinearity in structural dynamics: detection, identification and modelling. Bristol, UK: IOP.

[4] Schoukens J, Pintelon R, Rolain Y 2012 Mastering system identification in 100 exercises. New York, NY: Wiley-IEEE Press.

[5] Petrov E, Ewins D 2004 State-of-the-art dynamic analysis for nonlinear gas turbine structures. J. Aerospace Eng. 218, 199–211. (10.1243/0954410041872906)

[6] Carella A, Ewins D 2011 Identifying and quantifying structural nonlinearities in engineering applications from measured frequency response functions. Mech. Syst. Signal Process. 25, 1011–1027. (10.1016/j.ymssp.2010.09.011)

[7] Vazquez-Feijoo J, Worden K, Stanway R 2004 System identification using associated linear equations. Mech. Syst. Signal Process. 18, 431–455. (10.1016/S0888-3270(03)00078-5)

[8] Vazquez Feijoo J, Worden K, Montes Garcia P, Lagunez Rivera L, Juarez Rodriguez N, Pech Perez A 2010 Analysis of MDOF nonlinear systems using associated linear equations. Mech. Syst. Signal Process. 24, 2824–2843. (10.1016/j.ymssp.2010.04.008)

[9] Atkins P, Wright J, Worden K 2000 An extension of force appropriation to the identification of non-linear multi-degree-of-freedom systems. J. Sound Vib. 237, 23–43. (10.1006/jsvi.2000.3033)

[10] Peeters M, Kerschen G, Golinval J 2011 Modal testing of nonlinear vibrating structures based on nonlinear normal modes: experimental demonstration. Mech. Syst. Signal Process. 25, 1227–1247. (10.1016/j.ymssp.2010.11.006)

[11] Worden K, Green P 2014 A machine learning approach to nonlinear modal analysis. In Dynamics of civil structures, vol. 4 (ed. Catbas FN), pp. 521–528. New York, NY: Springer (10.1007/978-3-319-04546-7_56)

[12] Masri S, Sassi H, Caughey T 1982 A nonparametric identification of nearly arbitrary nonlinear systems. J. Appl. Mech. 49, 619–628. (10.1115/1.3162537)

[13] Crawley E, O’Donnell K 1986 Identification of nonlinear system parameters in joints using the force-state mapping technique. In 27th Structures, Structural Dynamics and Materials Conf., pp. 659–667. AIAA paper 86–1013. (10.2514/6.1986-1013)

[14] Feldman M 2011 Hilbert transform applications in mechanical vibration. New York, NY: Wiley.

[15] Delprat N, Escudie B, Guillemain P, Kronland-Martinet R, Tchamitchian P, Torresani B 1992 Asymptotic wavelet and Gabor analysis: extraction of instantaneous frequencies. IEEE Trans. Inform. Theory 38, 644–664. (10.1109/18.119728)

[16] Cohen L 1995 Time-frequency analysis. Englewood Cliffs, NJ: Prentice Hall.

[17] Newland D 1989 Mechanical vibration analysis and computation. New York, NY: Dover.

[18] Huang N, Shen Z, Long S, Wu M, Shih H, Zheng Q, Yen N-C, Tung C, Liu H 1998 The empirical mode decomposition and the Hilbert spectrum for nonlinear and non-stationary time series analysis. Proc. R. Soc. Lond. A 454, 903–995. (10.1098/rspa.1998.0193)

[19] Spina D, Valente C, Tomlinson G 1996 A new procedure for detecting nonlinearity from transient data using the Gabor transform. Nonlinear Dyn. 11, 235–254. (10.1007/BF00120719)

[20] Staszewski W 2000 Analysis of non-linear systems using wavelets. J. Mech. Eng. Sci. 214, 1339–1353. (10.1243/0954406001523317)

[21] Royston T, Singh R 1996 Periodic response of mechanical systems with local non-linearities using an enhanced Galerkin technique. J. Sound Vib. 194, 243–263. (10.1006/jsvi.1996.0355)

[22] Kitada Y 1998 Identification of nonlinear structural dynamic systems using wavelets. J. Eng. Mech. 124, 1059–1066. (10.1061/(ASCE)0733-9399(1998)124:10(1059))

[23] Yasuda K, Kawamura S, Watanabe K 1988 Identification of nonlinear multi-degree-of-freedom systems (identification under noisy measurements). Japan Soc. Mech. Eng. 31, 502–509.

[24] Mohammad K, Worden K, Tomlinson G 1992 Direct parameter estimation for linear and non-linear structures. J. Sound Vib. 152, 471–499. (10.1016/0022-460X(92)90482-D)

[25] Rice H, Fitzpatrick J 1991 A procedure for the identification of linear and non-linear multi-degree-of-freedom systems. J. Sound Vib. 149, 397–411. (10.1016/0022-460X(91)90444-O)

[26] Adams D, Allemang R 2000 A frequency domain method for estimating the parameters of a non-linear structural dynamic model through feedback. Mech. Syst. Signal Process. 14, 637–656. (10.1006/mssp.2000.1292)

[27] Richards C, Singh R 1998 Identification of multi-degree-of-freedom non-linear systems under random excitation by the ‘reverse path’ spectral method. J. Sound Vib. 213, 673–708. (10.1006/jsvi.1998.1522)

[28] Muhamad P, Sims N, Worden K 2012 On the orthogonalised reverse path method for nonlinear system identification. J. Sound Vib. 331, 4488–4503. (10.1016/j.jsv.2012.04.034)

[29] Zimmerman D, Hasselman T, Anderson M 2005 Approximation and calibration of nonlinear structural dynamics. Nonlinear Dyn. 39, 113–128. (10.1007/s11071-005-1917-x)

[30] Peng Z, Lang Z, Billings S 2007 Crack detection using nonlinear output frequency response functions. J. Sound Vib. 201, 777–788. (10.1016/j.jsv.2006.10.039)

[31] Noel J, Kerschen G 2013 Frequency-domain subspace identification for nonlinear mechanical systems. Mech. Syst. Signal Process. 40, 701–717. (10.1016/j.ymssp.2013.06.034)

